# *Hoplatessara luxuriosa* (Silvestri, 1895) (Diplopoda, Polydesmida, Paradoxosomatidae) is native to Australia, not New Guinea

**DOI:** 10.3897/zookeys.329.5976

**Published:** 2013-09-05

**Authors:** Robert Mesibov, Catherine A. Car

**Affiliations:** 1 Queen Victoria Museum and Art Gallery, 2 Invermay Road, Launceston, Tasmania 7248, Australia; 2 Department of Terrestrial Zoology, Western Australian Museum, Locked Bag 49, Welshpool DC, Western Australia 6986, Australia

**Keywords:** Diplopoda, Polydesmida, Paradoxosomatidae, New South Wales, Australia, L.M. D’Albertis

## Abstract

*Hoplatessara luxuriosa* (Silvestri, 1895) is partly redescribed and illustrated. Its native range is shown to be in the cool-climate uplands of New South Wales, Australia. *H. luxuriosa* was originally labelled as collected by L.M. D’Albertis at Sorong in New Guinea. D’Albertis collected on Sorong Island in 1872 and spent the following year in Sydney, New South Wales, before returning to Europe with his New Guinea specimens. It is possible that D’Albertis himself collected *H. luxuriosa* in 1873, and that the mislabelling occurred later.

## Introduction

The native range of the millipede *Hoplatessara luxuriosa* (Silvestri, 1895) has long been uncertain. It was first described as *Strongylosoma luxuriosum* by Silvestri (1895) from a male collected by L.M. D’Albertis at Sorong in New Guinea (now in Indonesia’s West Papua province) and deposited in the Museo Civico di Storia Naturale, Genoa, Italy.

Jeekel (1956, 1967, 1968, 1984, 2003) doubted this record, as all other species assigned to *Hoplatessara* Verhoeff, 1928 had been collected in Australia. Jeekel (1956, 1968) suggested that Silvestri’s material had been mislabelled, and that *Hoplatessara luxuriosa* ‘will be found sooner or later to occur in New South Wales’ (Jeekel 2003, p. 28).

Car (2009) listed *Hoplatessara luxuriosa* as a New South Wales native on the basis of specimens collected in that State and deposited in the Australian Museum. Here we give details of those and more recent collections, make minor additions to the excellent redescriptions by Jeekel (1956, 1967), and suggest a possible reason for the ‘Sorong’ confusion.

## Materials and methods

**Figures 1–2.**
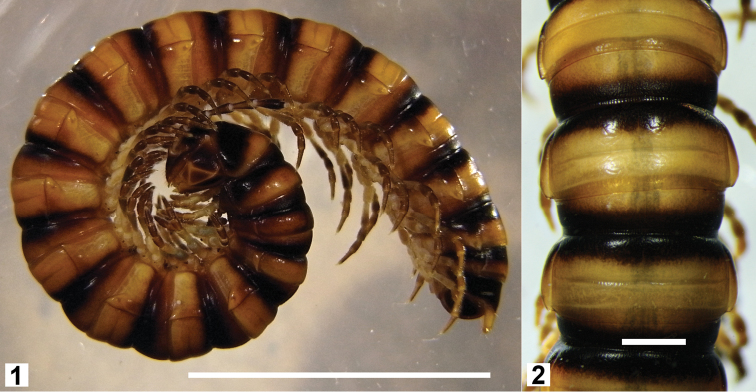
*Hoplatessara luxuriosa* (Silvestri, 1895), female ex AM KS.120531. Whole animal (**1**, scale bar = 10 mm) and dorsal view of midbody rings (**2**, scale bar = 1 mm).

**Figure 3–4.**
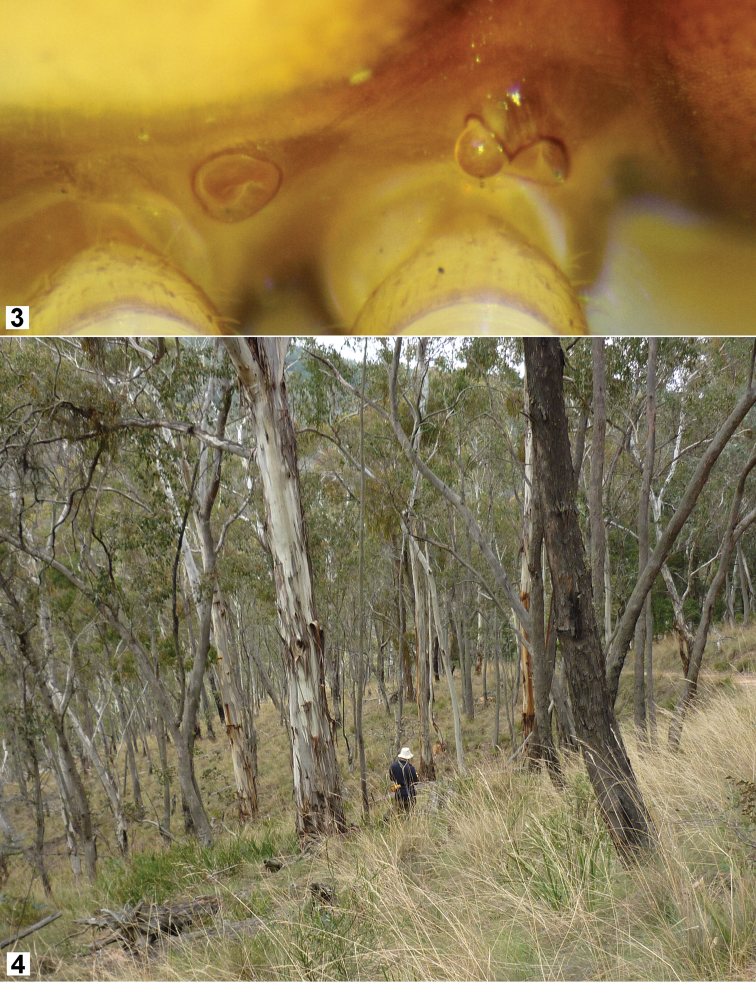
**3** *Hoplatessara luxuriosa* (Silvestri, 1895), female ex AM KS.120531, right lateral view of midbody spiracles (anterior to right) **4** Grassy forest habitat of *Hoplatessara luxuriosa* (Silvestri, 1895) along Daylight Creek Road near Sunny Corner, NSW, on 30 April 2013.

**Figures 5–8.**
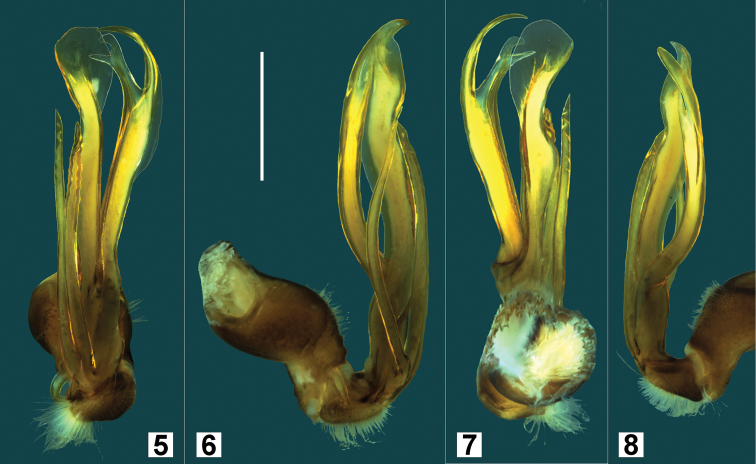
*Hoplatessara luxuriosa* (Silvestri, 1895), male ex AM KS.120532. Posterior (**5**), medial (**6**), anterior (**7**) and lateral (**8**) views of left gonopod, not to same scale. Scale bar in Fig. **6** = 1 mm.

Specimens are stored in ethanol in the Australian Museum. Fig. 1 was taken with a Ricoh GX200 and Figs 2 and 3 with a Canon EOS 1000D digital SLR camera mounted on a Nikon SMZ800 binocular dissecting microscope equipped with a beam splitter. Figs 1-3 are manually stacked composites processed with Zerene Stacker 1.04. Figs 5-8 were generated with a Leica MZ16A automontage imaging system using Leica Application Suite Version 3.7.0. Final figures were prepared using GIMP 2.8 image editing software. The latitude/longitude datum for collection details is WGS84. Abbreviations: AM = Australian Museum, Sydney, Australia; MCG = Museo Civico di Storia Naturale, Genoa, Italy; NSW = New South Wales, Australia.

## Results

### Order Polydesmida Pocock, 1887
Suborder Strongylosomatidea Brölemann, 1916
Family Paradoxosomatidae Daday, 1889
Subfamily Australiosomatinae Brölemann, 1916
Tribe Australiosomatini Brölemann, 1916

#### Hoplatessara luxuriosa

(Silvestri, 1895)

http://species-id.net/wiki/Hoplatessara_luxuriosa

Figs 1 –3 , 5–8

Strongylosoma luxuriosum Silvestri 1895: 643.
Strongylosoma luxuriosum Attems 1898: 318.
Strongylosoma luxuriosum Attems 1914: 221.
Strongylosoma (?) *luxuriosum*Attems 1915: 22.
Strongylosoma luxuriosum Chamberlin 1920: 119.
Antichiropus luxuriosus Attems 1937: 271. [comb. n.]
Hoplatessara luxuriosum [sic] Jeekel 1956: 184; figs 1–4, p. 186. [comb. n.]
Hoplatessara luxuriosa Jeekel 1967: 377. [Lectotype chosen]
Hoplatessara luxuriosa Jeekel 1968: 23, 25.
Hoplatessara luxuriosa Jeekel 1981: 51.
Hoplatessara luxuriosa Jeekel 1984: 38, 43.
Hoplatessara luxuriosa Jeekel 2003: 28.

##### Lectotype.

Male, lacking segments 8 and 9, Sorong, New Guinea [see Discussion], L.M. D’Albertis, MCG.

##### Paralectotypes.

1 female, 1 female fragment, 1 male fragment, same details, MCG.

##### Material examined.

1 male, Sunny Corner State Forest near Bathurst, NSW, 33°24'S, 149°51'E ±2 km, 4 December 1972, J.S. Disney, under pine log, AM KS.18542; 1 male, near Merrill, SW of Crookwell, NSW [locality text not on label], 34°40'S, 149°17'E ±2 km, 20 April 1990, [collector uncertain], AM KS.106310; 6 males, 2 females, 1 juvenile, 5 km NE of Colo, NSW [locality text not on label], 33°45'S, 149°17'E ±2 km, 653 m a.s.l., 20 April 1990, L. Kirwan, AM KS.106320; 2 males, Sunny Corner near Bathurst, NSW, 33°24'S, 149°53'E ±2 km, 24-27 January 1997, S.J. Fellenberg, AM KS.96088; 1 male, 1 female, 1 stadium 7 female, Daylight Creek Road near Sunny Corner, NSW, 33°21'51"S, 149°53'39"E ±100 m, 1050 m a.s.l., 30 April 2013, R. Mesibov and T. Moule, AM KS.120531; 1 male, 1 female, Sunny Corner Road near Sunny Corner, NSW, 33°23'58"S, 149°54'22"E ±25 m, 1210 m a.s.l., same date and collectors, AM KS.120532.

##### Description.

Jeekel (1956, 1967) gave admirably complete redescriptions of the specimens examined by Silvestri (1895), and here we add only a few details:

Live and freshly preserved males and females (Figs 1, 2) with pale brownish-yellow ground colour, lighter ventrally; darker brown on prozonites and anterior portion of metazonites, darkest at waist and dorsally; head dark brown dorsally, lightening ventrally; collum dark brown ringed with pale brownish-yellow; antennae dark brown, lighter basally; legs with coxae, prefemur and basal portion of femur pale yellow, and postfemur, tibia and tarsus brown, darkening distally; preanal ring dark brown with pale yellow epiproct; hypoproct light brown, anal valves dark brown ringed with pale brownish-yellow.

Small pleural keels on female rings 2–4, more prominent on rings 3 and 4; traces of keels on male rings 2–4.

Male ring 6 sternite with transverse brushes of long setae between legpairs 6 and 7. Male ring 5 sternite with sparse transverse brush of long setae between legpair 5, well-separated from sternal lamella between legpair 4.

Spiracles on diplosegments of males and females well-separated (Fig. 3); posterior spiracle crater-like, anterior spiracle rim oval (long axis more or less dorsoventral), dorsal portion of rim extended posterolaterally around emergent, finely textured, subspherical spiracle.

Gonopods (Figs 5–8) as described by Jeekel (1956) and illustrated in posterior view (Fig. 4 in Jeekel 1956); the slight bulge on the medial side of the femoral process is partly obscured in posterior view (Fig. 5) and is shown more clearly in anterior view (Fig. 7).

##### Distribution and habitat.

**Figure 9.**
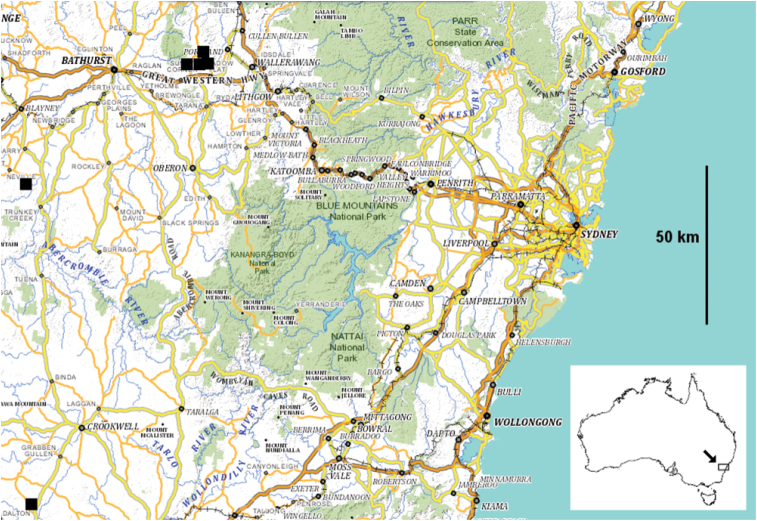
Localities for *Hoplatessara luxuriosa* (Silvestri, 1895) as of 30 April 2013 (black squares). Inset shows location of main map. Base map from the Spatial Information Exchange, New South Wales Department of Finance and Services (http://maps.six.nsw.gov.au/ [accessed 9 July 2013]), showing named localities mentioned in text.

Occurs in the high country west of the Blue Mountains in New South Wales (Fig. 9) in grassy eucalypt forest (Fig. 4) and plantations of *Pinus radiata*, where adults were found in 2013 sheltering under logs and small pieces of fallen wood or bark. We have not yet confirmed by further collecting the two 1990 localities south of Bathurst (Fig. 9); if these are correct, the north-south range of *Hoplatessara luxuriosa* is ca 150 km.

##### Remarks.

The structure of the anterior spiracle on diplosegments (Fig. 3) is very similar to that found in other australiosomatine paradoxosomatids [see figs 2A, 2B, 3C in Mesibov (2009)].

## Discussion

The known *Hoplatessara luxuriosa* range (Fig. 9) is in the cool, dry ‘Central Tablelands’ region of New South Wales.

Long-term climate averages for the town of Lithgow, ca 25 km southeast of the collection localities near Sunny Corner and ca 250 m lower in elevation, are 0.7/10.4°C (mean minimum/mean maximum) in the coldest month (July) and 11.9/25.5 in the warmest month (January), with a mean annual rainfall of 858 mm well-distributed through the year (Australian Bureau of Meterorology, http://www.bom.gov.au/climate/averages/tables/cw_063224.shtml [accessed 9 May 2013]). Frosts and light winter snowfalls are common events in the area.

In comparison, average night/day temperatures in Sorong, Indonesia are ca 25/31°C throughout the year, and the well-distributed annual rainfall is 2840 mm (http://www.weatherbase.com/weather/weather.php3?s=20579 [accessed 9 May 2013]). It seems highly unlikely that *Hoplatessara luxuriosa* could occur naturally in Sorong, or establish there if introduced. How, then, did the New South Wales specimens examined by Silvestri and Jeekel come to be labelled ‘Sorong’?

On his first expedition to New Guinea, the Italian naturalist and explorer L.M. D’Albertis arrived on Sorong Island (close to the coast and near the modern-day city of Sorong) at the end of April 1872 and based himself there until mid-July, collecting birds and insects (D’Albertis 1880). He returned to Sorong Island for several days in mid-November. D’Albertis arrived in Sydney on 1 February 1873 (D’Albertis 1880), took up residence in Double Bay and began a ten-month recovery from fevers and other ailments suffered during his first New Guinea expedition. He left Sydney on 20 December 1873, ‘not yet completely restored to health’ (D’Albertis 1880, vol. 1, p. 224), on a voyage ending in Italy (Gibbney 1972). His Sorong and other specimens became part of the collection at the newly established Museo Civico di Storia Naturale ‘Giacomo Doria’, where they were later examined by Silvestri.

It is possible that D’Albertis himself collected *Hoplatessara luxuriosa* during his long stay in New South Wales in 1873, and that the specimens were unintentionally mixed with those from Sorong Island, or kept separate and later mislabelled. The Main Western line of the New South Wales railways had reached the eastern edge of the known *Hoplatessara luxuriosa* range at Wallerawang in 1870 and Tarana in 1872 (http://www.nswrail.net/lines/show.php?name=NSW:main_west [accessed 11 May 2013]; see Fig. 9). Landscape photographs taken at Wallerawang ca 1871 (http://investigator.records.nsw.gov.au/asp/photosearch/photo.asp?17420_a014_a014001362 [accessed 11 May 2013]) and Tarana in the period 1870-1880 (http://nla.gov.au/nla.pic-vn5748868 [accessed 11 May 2013]) show open eucalypt forest, a habitat type known to be occupied by *Hoplatessara luxuriosa*. The recuperating D’Albertis could have travelled to either place from Sydney in the comfort of a passenger train in half a day.

D’Albertis returned to Sydney in 1876, 1877 and 1878 but did not revisit ‘Sorong’ during his four later New Guinea expeditions (D’Albertis 1880). We have not been able to locate any documents relating to D’Albertis’ movements in New South Wales during his time there in the 1870s, and our explanation for the ‘Sorong’ confusion remains speculative.

## Supplementary Material

XML Treatment for Hoplatessara luxuriosa
